# The chromatin remodeling factor Bap55 functions through the TIP60 complex to regulate olfactory projection neuron dendrite targeting

**DOI:** 10.1186/1749-8104-6-5

**Published:** 2011-02-01

**Authors:** Joy S Tea, Liqun Luo

**Affiliations:** 1 Howard Hughes Medical Institute, Department of Biology, Neurosciences Program, Stanford University, Stanford, CA 94305, USA

## Abstract

**Background:**

The *Drosophila *olfactory system exhibits very precise and stereotyped wiring that is specified predominantly by genetic programming. Dendrites of olfactory projection neurons (PNs) pattern the developing antennal lobe before olfactory receptor neuron axon arrival, indicating an intrinsic wiring mechanism for PN dendrites. These wiring decisions are likely determined through a transcriptional program.

**Results:**

We find that loss of Brahma associated protein 55 kD (Bap55) results in a highly specific PN mistargeting phenotype. In *Bap55 *mutants, PNs that normally target to the DL1 glomerulus mistarget to the DA4l glomerulus with 100% penetrance. Loss of Bap55 also causes derepression of a GAL4 whose expression is normally restricted to a small subset of PNs. Bap55 is a member of both the Brahma (BRM) and the Tat interactive protein 60 kD (TIP60) ATP-dependent chromatin remodeling complexes. The *Bap55 *mutant phenotype is partially recapitulated by Domino and Enhancer of Polycomb mutants, members of the TIP60 complex. However, distinct phenotypes are seen in Brahma and Snf5-related 1 mutants, members of the BRM complex. The *Bap55 *mutant phenotype can be rescued by postmitotic expression of Bap55, or its human homologs BAF53a and BAF53b.

**Conclusions:**

Our results suggest that Bap55 functions through the TIP60 chromatin remodeling complex to regulate dendrite wiring specificity in PNs. The specificity of the mutant phenotypes suggests a position for the TIP60 complex at the top of a regulatory hierarchy that orchestrates dendrite targeting decisions.

## Background

The stereotyped organization of the *Drosophila *olfactory system makes it an attractive model to study wiring specificity. The first olfactory processing center is the antennal lobe, a bilaterally symmetric structure at the anterior of the *Drosophila *brain. It is composed of approximately 50 glomeruli in a three-dimensional organization. Each olfactory projection neuron (PN) targets its dendrites to one of those glomeruli to make synaptic connections with a specific class of olfactory receptor neurons. Each PN sends its axon stereotypically to higher brain centers [1-3].

During development, the dendrites of PNs pattern the antennal lobe prior to axons of olfactory receptor neurons [4]. The specificity of PN dendrite targeting is largely genetically pre-determined by the cell-autonomous action of transcription factors, several of which have been previously described [5-8]. Furthermore, chromatin remodeling factors have been shown to play an important role in PN wiring [8], although very little is currently known about their specific functions. We report here a genetic screen for additional factors that regulate PN dendrite wiring specificity, and identify Brahma associated protein 55 kD (Bap55) as a regulator of PN dendrite wiring specificity as part of the TIP60 chromatin remodeling complex.

**Figure 1:**
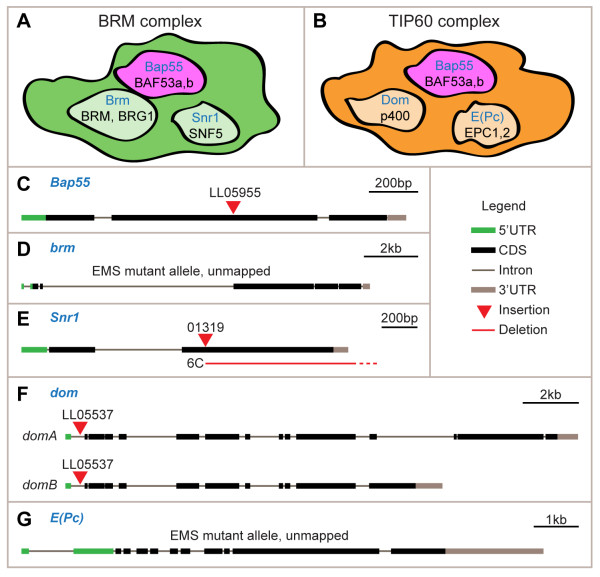
**Genes analyzed in this study**. **(A) **We analyze three components of the BRM complex in this study. In this schematic representation, *Drosophila *genes are labeled in blue and their human homologs are labeled in black. Shapes, sizes, and locations have no significance. Additional complex components are not shown. **(B) **We analyze three components of the TIP60 complex in this study. Symbols are as in (A). **(C) ***Bap55*^*-/- *^in this study denotes the LL05955 allele, which contains a piggyBac insertion in the coding sequence (CDS) [22]. **(D) ***brm*^*-/- *^denotes the *brm*^*2 *^allele, an ethylmethanesulfonate (EMS) mutant that is a protein null [27]. **(E) ***Snr1*^-^^/- ^denotes the *Snr1*^*6C *^allele, which is a deletion removing much of the *Snr1 *gene and extending into the next gene, *HDAC3*. *HDAC3 *mutants have been previously shown to have no phenotype in PNs [8]. Yet in this study we also expressed *UAS-HDAC3-3xFLAG *in the *Snr1*^*6C *^mutant PNs to account for any phenotypes due to *HDAC3 *deletion. We additionally analyzed the 01319 allele, a P-element insertion in the *Snr1 *coding sequence, which gave the same phenotypes as the *Snr1*^*6C *^allele (data not shown). **(F) ***dom*^*-/- *^denotes the LL05537 allele, which contains a piggyBac insertion in an intron upstream of the translational start. The piggyBac element contains splice acceptor sites and stop codons in all three coding frames [22], which likely disrupts all *dom *isoforms. **(G) ***E(Pc)*^*-/- *^denotes the *E(Pc)*^*1 *^allele, an EMS mutant in which *E(Pc)*^*1*^*/+ *heterozygous flies exhibit only 10 to 21% of the mRNA of wild-type flies. In principle, a null would be expected to have 50% [35]. Scale bars are provided for each panel.

Bap55 is an actin-related protein, the majority of which physically associates with the Brahma (BRM) chromatin remodeling complex in *Drosophila *embryo extracts [9] (Figure 1A). There are two distinct BRM complexes: BAP (Brahma associated proteins; homologous to yeast SWI/SNF) and PBAP (Polybromo-associated BAP; homologous to yeast RSC), both of which contain Brahma, Bap55, and Snf5-Related 1 (Snr1) [10]. The human homologs of the BAP and PBAP complexes are called the BAF (Brg1 associated factors) and PBAF (Polybromo-associated BAF) complexes, respectively. The BRM/BAF complexes are members of the SWI/SNF family of ATP-dependent chromatin-remodeling complexes, and have been shown to both activate and repress gene transcription, in some cases, of the same gene [11-14].

In *Drosophila*, RNA interference knockdown of *Bap55 *in embryonic class I da neurons revealed dendrite misrouting phenotypes and reduced arborization [15]. The human homologs of Bap55 are BAF53a and BAF53b, with approximately equal homology. BAF53a is important for maintaining embryonic stem cell self-renewal and pluripotency as a part of the BAF complex found in embryonic stem cells (esBAF) [16]. BAF53b is important for dendritic outgrowth as a part of the BAF complex found in postmitotic neurons (nBAF) [17]. However, previous experiments have not clearly distinguished whether Bap55/BAF53b acts exclusively as a part of the BRM/BAF complex in regulating dendrite development.

In experiments purifying proteins in complex with tagged *Drosophila *Pontin in S2 cells, Bap55 was also co-purified as a part of the TIP60 complex, as determined by mass spectrometry [18] (Figure 1B). The TIP60 histone acetyltransferase complex has been shown to be involved in many processes, including both transcriptional activation and repression [19]. The complex contains many components, including Bap55, Domino (Dom), and Enhancer of Polycomb (E(Pc)) [18]. Dom, homologous to human p400, is the catalytic DNA-dependent ATPase; its ATPase domain is highly similar to *Drosophila *Brahma and human BRG1 ATPase domains [20]. E(Pc) is homologous to human EPC1 and EPC2 and is an unusual member of the Polycomb group; it does not exhibit homeotic transformations on its own, but rather enhances mutations in other Polycomb group genes [21].

We provide evidence that Bap55 functions as a part of the TIP60 complex rather than the BRM complex in postmitotic PNs to control their dendrite wiring specificity.

## Results

### Bap55 is required in projection neurons for dendrite targeting

**Figure 2:**
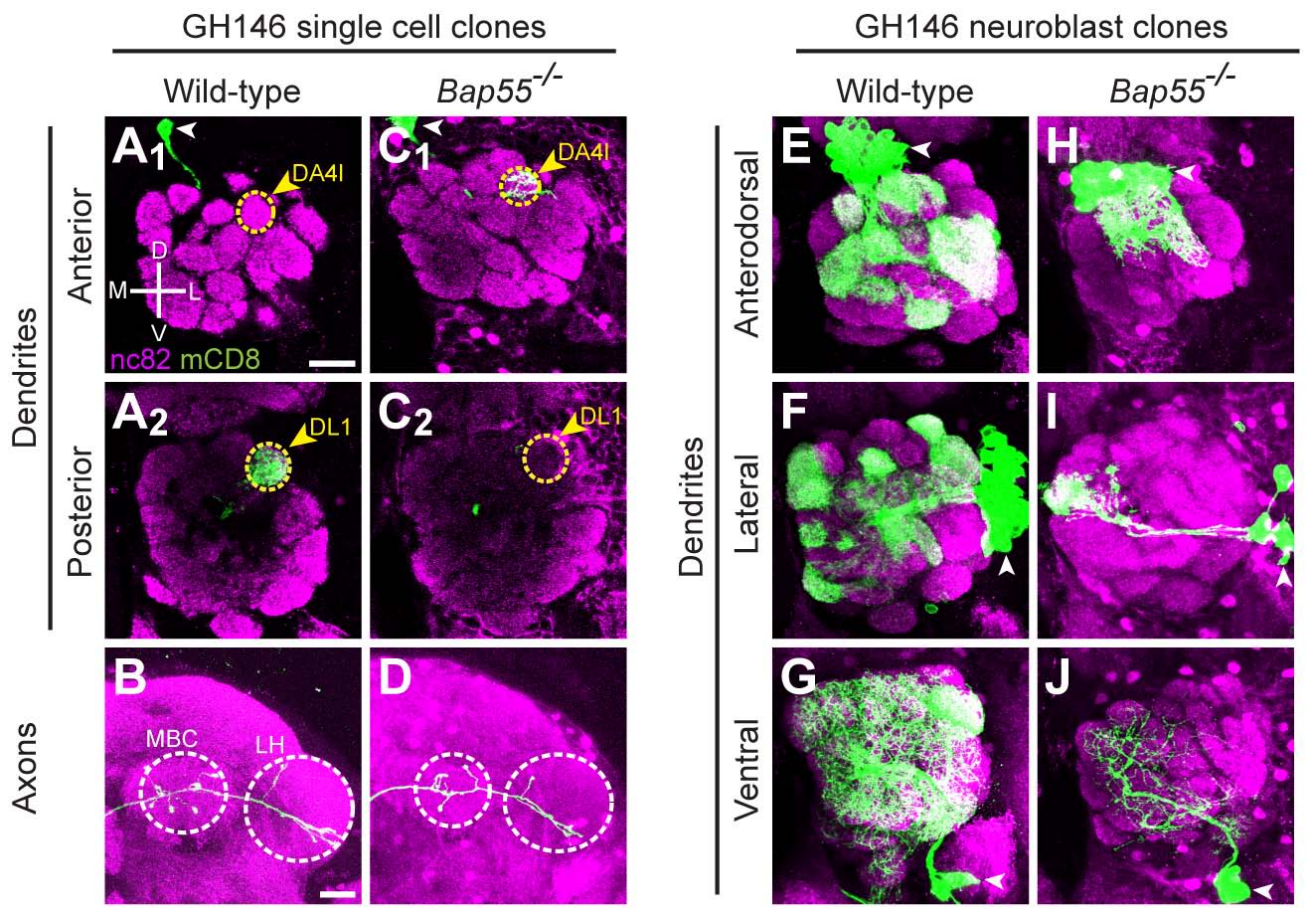
**Bap55 regulates projection neuron dendrite targeting**. **(A) **Wild-type (WT) DL1 PN dendrites target specifically to the posterior glomerulus DL1 (yellow dashed circle in A_2_) and never target to the anterior glomerulus DA4l (yellow dashed circle in A_1_). White arrowhead denotes cell body in all images. **(B) **WT DL1 axons show an L-shaped pattern in the lateral horn (LH), with branches in the mushroom body calyx (MBC; both outlined with white dashed circles). **(C) ***Bap55*^*-/- *^PN dendrites fully mistarget to the anterior glomerulus DA4l (C_1_), abandoning the posterior glomerulus DL1 (C_2_). **(D) ***Bap55*^*-/- *^PN axons show the stereotypical L-shaped pattern in the LH with branches in the MBC. **(E) **WT GH146 anterodorsal neuroblast clones are characterized by their cell body location dorsal to the antennal lobe (white arrowhead in all images). The dendrites target to stereotyped glomeruli in the antennal lobe. **(F) **WT GH146 lateral neuroblast clones are characterized by their cell body location lateral to the antennal lobe with dendrites targeting to stereotyped glomeruli. **(G) **WT GH146 ventral neuroblast clones are characterized by their cell body location ventral to the antennal lobe with dendrites targeting across the antennal lobe. **(H, I) ***Bap55*^*-/- *^GH146-labeled anterodorsal (H) and lateral (I) neuroblast clones maintain their cell body location dorsal and lateral to the antennal lobe, respectively. Yet their dendrites do not target proper glomeruli. **(J) ***Bap55*^*-/- *^GH146-labeled ventral neuroblast clones maintain their cell body location ventral to the antennal lobe, yet their dendrites target a small area of the antennal lobe. Green marks mCD8-GFP-labeled PNs generated by MARCM and labeled using GH146-GAL4. (A, C) show partial confocal stacks; (B, D, E-J) show full confocal stacks; magenta is the presynaptic marker nc82; extraneous magenta punctate staining outside of the antennal lobe in some panels is dsRed background from the piggyBac and/or GH146-GAL4 insertions. Scale bars: 20 μm in (A) (for A, C, E-J) and (B) (for B, D).

To further our understanding of dendrite wiring specificity in *Drosophila *olfactory PNs, we performed a MARCM-based forward genetic screen using piggyBac insertional mutants [22]. MARCM allows visualization and genetic manipulation of single cell or neuroblast clones in an otherwise heterozygous background, permitting the study of essential genes in mosaic animals [23]. In this screen, we used GH146-GAL4 to label a single PN born soon after larval hatching [1], which in wild-type (WT) animals always projects its dendrites to the dorsolateral glomerulus DL1 in the posterior of the antennal lobe (Figure 2A). The DL1 PN also exhibits a stereotyped axon projection, forming an L-shaped pattern in the lateral horn, with additional branches in the mushroom body calyx (Figure 2B). We identified a mutant, called LL05955, in which DL1 PNs mistargeted to the dorsolateral glomerulus DA4l in the anterior of the antennal lobe (Figure 2C). This phenotype is strikingly specific, with 100% penetrance (Table 1). Arborization of mutant axons, however, was not obviously altered (Figure 2D). We identified the piggyBac insertion site using inverse PCR [22] and Splinkerette PCR [24]. LL05955 is inserted into the coding sequence of *Bap55 *(Figure 1C), encoding a homolog of human BAF53a and BAF53b. Precise excision of the piggyBac insertion reverted the dendrite mistargeting phenotype, confirming that disruption of the *Bap55 *gene causes the dendrite mistargeting (Table 1).

**Table 1:** Discrete mistargeting of TIP60 complex components

Genotype	Total n	%DL1	%DA4l	%DL1 +DA4l	%DM6	%DA4l + DM6	%DA2	%DA2 + DM6
WT	25	**100**	0	0	0	0	0	0
*Bap55*^*-/-*^	28	0	**100**	0	0	0	0	0
*Bap55*^*-/- *^*precise excision*	19	**100**	0	0	0	0	0	0
*Bap55*^*-/- *^*+UAS-Bap55*	32	**90**	0	0	10	0	0	0
*UAS-Bap55*	16	**100**	0	0	0	0	0	0
*Bap55*^*-/- *^*+UAS-BAF53a*	19	**68**	0	0	32	0	0	0
*Bap55*^*-/- *^*+UAS-BAF53b*	21	**94**	0	0	6	0	0	0
*dom*^*-/-*^	23	4	9	**87**	0	0	0	0
*dom*^*-/- *^*precise excision*	18	**100**	0	0	0	0	0	0
*dom*^*-/- *^*+UAS-Bap55*	15	27	6	**67**	0	0	0	0
*E(Pc)*^*-/-*^	22	5	**45**	0	14	0	36	0
*E(Pc)*^*-/- *^*+UAS-Bap55*	20	0	0	0	0	**70**	5	25

Shown is the total number of analyzed PN single cell clones and percent targeting to listed glomeruli. In bold is the glomerulus with the highest percentage of targeting.

In addition to causing DL1 mistargeting, *Bap55 *mutants also display neuroblast clone phenotypes. In WT, GH146-GAL4 can label three distinct types of PN neuroblast clones generated in newly hatched larvae. Two of these clones, the anterodorsal neuroblast clone (Figure 2E) and the lateral neuroblast clone (Figure 2F), possess cell bodies that lie dorsal or lateral to the antennal lobe, respectively. PNs from these two lineages project their dendrites to stereotyped and nonoverlapping subsets of glomeruli in the antennal lobe. The third type of clone, the ventral neuroblast clone, has cell bodies that lie ventral to the antennal lobe and dendrites that target throughout the antennal lobe due to the inclusion of at least one PN that elaborates its dendrites to all glomeruli (Figure 2G) [1,2].

In *Bap55*^*-/- *^PNs, anterodorsal neuroblast clones display a mild reduction in cell number, and their dendrites are abnormally clustered in the anterior dorsal region of the antennal lobe, including the DA4l glomerulus (Figure 2H). Lateral neuroblast clones display a severe reduction in cell number, and the remaining dendrites are unable to target to many glomeruli throughout the antennal lobe (Figure 2I). Ventral neuroblast clones display a mild reduction in cell number and a reduced dendrite mass throughout the antennal lobe (Figure 2J). During development, the lateral neuroblast first gives rise to local interneurons before switching to produce PNs [25]; in mutants affecting cell proliferation, this property of the lateral neuroblast displays as a severe reduction in GH146-labeled PNs. The severely reduced cell number in *Bap55 *mutants suggests that Bap55 is essential for neuroblast proliferation or neuronal survival. In the anterodorsal and ventral neuroblasts, PN numbers are not drastically changed; thus, the phenotypes indicate that Bap55 is important for dendrite targeting in multiple classes of PNs.

### *Bap55 *mutants cause derepression of a PN-GAL4

**Figure 3:**
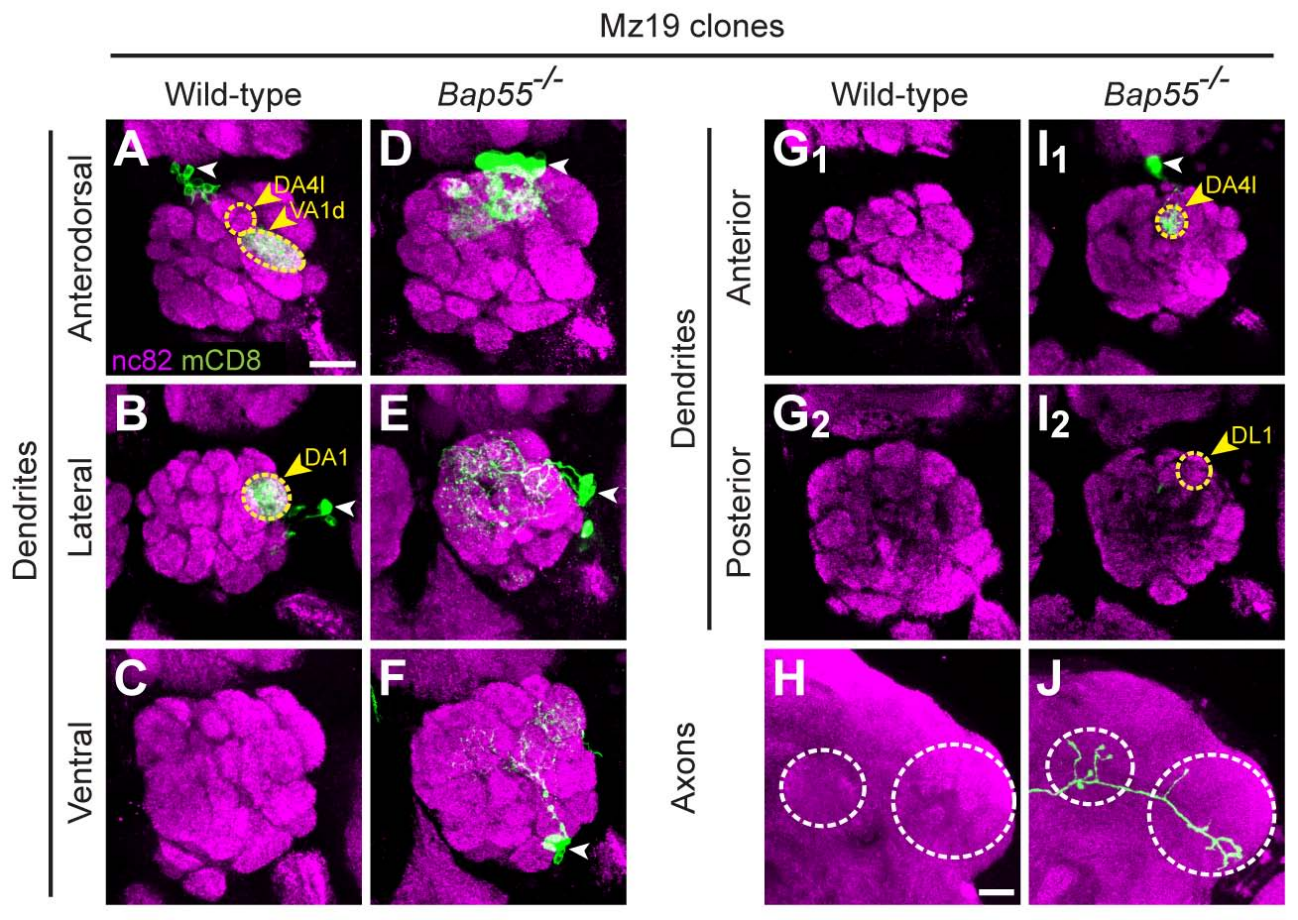
***Bap55 *mutants cause derepression of a PN-GAL4**. **(A) **WT Mz19 anterodorsal neuroblast clones label PNs targeting to the VA1d and DC3 glomeruli. The DC3 glomerulus is difficult to visualize in confocal stacks. **(B) **WT Mz19 lateral neuroblast clones label a single class of PNs targeting to the DA1 glomerulus. **(C) **WT Mz19 never labels ventral PN neuroblast clones. No labeling is observed in the antennal lobe. **(D) ***Bap55*^*-/- *^Mz19 anterodorsal neuroblast clones exhibit ectopic labeling of PNs targeting many glomeruli in the anterior antennal lobe. **(E) ***Bap55*^*-/- *^Mz19 lateral neuroblast clones exhibit ectopic labeling of local interneurons, which target throughout the antennal lobe. **(F) ***Bap55*^*-/- *^Mz19 clones ectopically label ventral PNs, which are never labeled in WT, and that target to very few glomeruli in the antennal lobe. **(G, H) **WT Mz19 never labels a single anterodorsal PN when clones are generated just after larval hatching. (G_1_, G_2_) show no labeling in the antennal lobe. (H) shows no axon labeling in the lateral horn. **(I, J) ***Bap55*^*-/- *^Mz19 exhibits ectopic labeling of single anterodorsal PNs, which target to the anterodorsal glomerulus DA4l (I_1_), while avoiding the posterior glomerulus DL1 (I_2_). The axons form an L-shaped pattern in the lateral horn, with branches in the mushroom body calyx (J). Green marks mCD8-GFP-labeled PNs generated by MARCM and labeled using Mz19-GAL4. (G, I) show partial confocal stacks; (A-F, H, J) show full confocal stacks; magenta is the presynaptic marker nc82; symbols are as in Figure 2. Scale bars: 20 μm in (A) (for A-G, I) and (H) (for H, J).

In WT, Mz19-GAL4 labels a subset of the GH146-GAL4 labeling pattern. It labels a small number of PNs derived from two neuroblasts, which can be clearly identified in WT clones generated in newly hatched larvae. Anterodorsal neuroblast clones target their dendrites to the VA1d glomerulus (Figure 3A), as well as the DC3 glomerulus residing immediately posterior to VA1d (not easily visible in confocal stacks). Lateral neuroblast clones target all dendrites to the DA1 glomerulus (Figure 3B). Unlike GH146-GAL4, WT Mz19-GAL4 never labels ventral neuroblast clones because it is not normally expressed in those cells (Figure 3C).

In *Bap55 *mutant PN clones, however, Mz19-GAL4 labels additional PNs in anterodorsal, lateral, and ventral clones (Figures 3D-F) compared to their WT counterparts (Figures 3A-C). This phenotype suggests that some Mz19-negative PNs now express Mz19-GAL4. In anterodorsal clones, Mz19-GAL4 labels additional cells, although not as many as are labeled by GH146-GAL4. The PNs also mistarget their dendrites to the anterior antennal lobe (Figure 3D), similar to mutant GH146-GAL4 anterodorsal neuroblast clones (Figure 2H). WT lateral neuroblast clones normally contain GH146-positive PNs and GH146-negative local interneurons [25]. In *Bap55*^*-/- *^lateral neuroblast clones, Mz19-GAL4 predominantly labels local interneurons that send their processes to many glomeruli throughout the antennal lobe (Figure 3E) and do not send axon projections to higher brain centers. Lateral clones also show ectopic PN labeling with a lower frequency (data not shown). The *Bap55 *mutant appears to cause derepression of Mz19-GAL4, resulting in labeled local interneurons. Ventral neuroblast clones are never labeled in WT Mz19-GAL4 (Figure 3C), yet are labeled in *Bap55 *mutants (Figure 3F). This further indicates a derepression of the Mz19-GAL4 labeling pattern.

Unlike GH146-GAL4, WT Mz19-GAL4 never labels single cell clones when clone induction is performed in newly hatched larvae (Figure 3G, H). This is because Mz19-GAL4 is not expressed in the DL1 PN, the only GH146-positive cell generated during this heat shock time of clone generation. However, in *Bap55 *mutant PN clones, Mz19-GAL4 ectopically labels single cell anterodorsal PN clones targeting to the DA4l glomerulus (Figure 3I), which show an L-shaped pattern in the lateral horn with branches in the mushroom body calyx (Figure 3J), similar to GH146-GAL4 labeling (Figures 2C, D). The simplest interpretation is that this compound phenotype reflects first a derepression of Mz19-GAL4 in the DL1 PN, and second a DL1 to DA4l mistargeting phenotype in *Bap55 *mutants.

### Bap55 is required in postmitotic PNs for dendrite targeting

**Figure 4:**
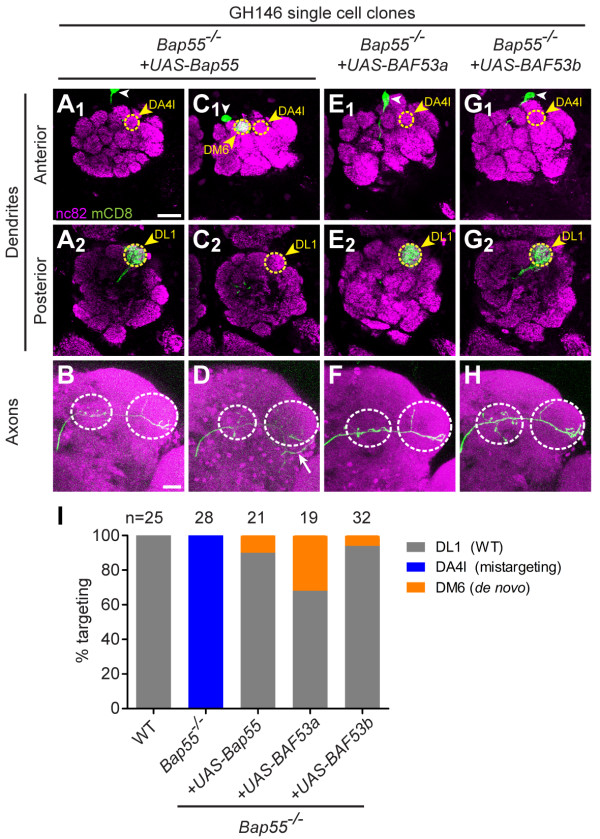
**Bap55 acts postmitotically in PNs and dendrite mistargeting can be suppressed by human BAF53a and b**. **(A, B) **Postmitotic expression of *UAS-Bap55 *can rescue the *Bap55*^*-/- *^dendrite mistargeting phenotype. The PN no longer targets to the anterior glomerulus DA4l (A_1_), but to the posterior glomerulus DL1 (A_2_). The L-shaped axon pattern is not altered (B). **(C, D) **In a small number of cases, postmitotic expression of *UAS-Bap55 *in a *Bap55*^*-/- *^single cell causes a *de novo *phenotype. The dendrites target to the anterior and medial glomerulus DM6 (C_1_), abandoning both DA4l (C_1_) and DL1 (C_2_). In these cases, it also causes a *de novo *axon phenotype of mistargeting ventrally to the lateral horn (white arrow in (D)). **(E, F) **Postmitotic expression of human *UAS-Baf53a *also rescues the *Bap55*^*-/- *^phenotype (E) and does not alter the L-shaped axon pattern (F). **(G, H) **Postmitotic expression of human *UAS-Baf53b *also rescues the *Bap55*^*-/- *^phenotype (G) and does not alter the L-shaped axon pattern (H). **(I) **Quantification of mistargeting phenotypes for (A, C, E, G). Green marks mCD8-GFP-labeled PNs generated by MARCM and labeled using GH146-GAL4. (A, C, E, G) show partial confocal stacks; (B, D, F, H) show full confocal stacks; magenta is the presynaptic marker nc82; symbols are as in Figure 2. Scale bars: 20 μm in (A) (for A, C, E, G) and (B) (for B, D, F, H).

To test whether Bap55 functions in the neuroblast or postmitotically in PNs, we used GH146-GAL4, which expresses only in postmitotic PNs [7], to express *UAS-Bap55 *in a *Bap55*^*-/- *^single cell clone. We show that the dendrite mistargeting phenotype is rescued to the WT DL1 glomerulus (Figure 4A, Table 1) and conclude that Bap55 functions postmitotically to regulate PN dendrite targeting. The axon phenotype remains the stereotypical L-shaped pattern (Figure 4B).

However, in 2 out of 21 cases, expression of *UAS-Bap55 *in a *Bap55*^*-/- *^single cell clone resulted in a *de novo *phenotype. The PN dendrites targeted to neither the DA4l nor the DL1 glomeruli, but to the DM6 glomerulus in the anterior medial region of the antennal lobe (Figure 4C, Table 1). In addition, the axon showed a mistargeting phenotype, extending ventrally to the lateral horn (Figure 4D). The two cases showed correlated DM6 dendrite and ventral axon mistargeting; the remaining 19 out of 21 cases showed full DL1 rescue and an L-shaped axon pattern. Expression of *UAS-Bap55 *in a WT single cell clone, however, did not cause any phenotype (n = 16; Table 1).

### Human BAF53a and b can rescue *Bap55 *mutant phenotypes

The *Drosophila *Bap55 protein is 70% similar and 54% identical to human BAF53a and 71% similar and 55% identical to human BAF53b. BAF53a and b are 91% similar and 84% identical to each other. Using GH146-GAL4 to express human BAF53a or b in a *Bap55*^*-/- *^single cell clone, we found that the human homologs can effectively rescue the *Bap55*^*-/- *^dendrite mistargeting phenotype (Figures 4E-H). Interestingly, both also cause the *de novo *DM6 dendrite and ventral axon mistargeting phenotypes in 6 out of 19 cases for BAF53a and 2 out of 32 cases for BAF53b. These phenotypes are quantified in Figure 4I and summarized in Table 1. Thus, human BAF53a and b can largely replace the function of *Drosophila *Bap55 in PNs.

### Mutations in other BRM complex components have distinct PN dendrite targeting phenotypes

To address whether Bap55 functions as a part of the BRM complex in PN dendrite targeting, we tested two additional BRM complex mutants for their PN dendrite phenotypes. We first tested Brahma (brm), the catalytic ATPase subunit of the BRM complex, which is required for the activation of many homeotic genes in *Drosophila *[26] (Figure 1D). Null mutations have been shown to decrease cell viability and cause peripheral nervous system defects [27]. RNA interference knockdown of *brm *in embryonic class I da neurons revealed dendrite misrouting phenotypes, although not identical to the *Bap55 *phenotype [15]. The human homologs of brm, BRM and BRG1 (Brahma-related gene-1), both have DNA-dependent ATPase activity. Inactivation of BRM in mice does not yield obvious neural phenotypes, but inactivation of BRG1 in neural progenitors results in reduced proliferation. BRG1 is likely to be required for various aspects of neural development, including proper neural tube development [28].

**Figure 5:**
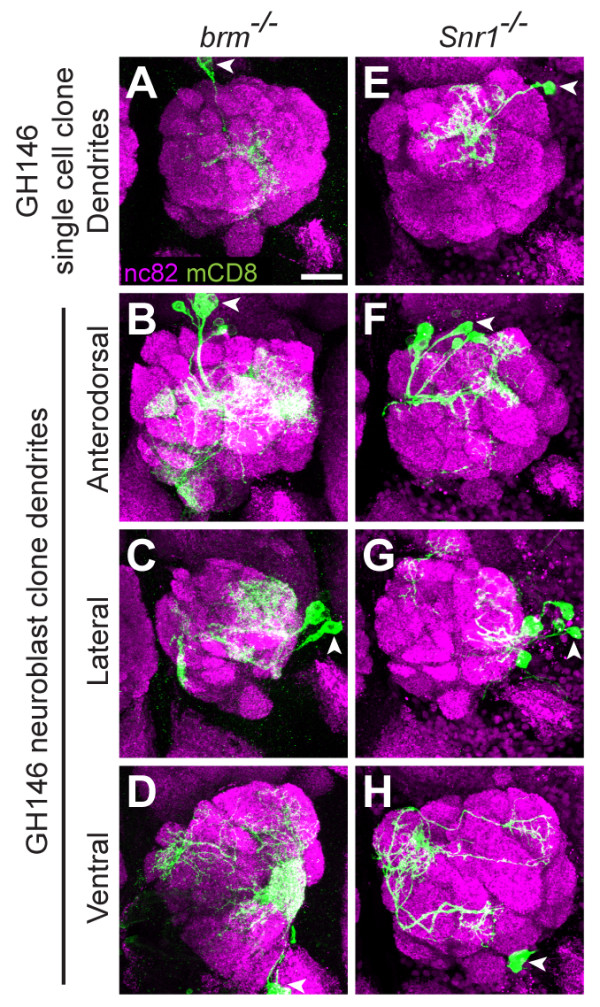
**Other BRM complex component mutants do not exhibit the same phenotypes as *Bap55 *mutants**. **(A) **Dendrites of *brm*^*-/- *^DL1 PNs mistarget to non-stereotyped areas of the antennal lobe. **(B, C) ***brm*^*-/- *^anterodorsal (B) and lateral (C) neuroblast clone PNs exhibit perturbed cell morphology, weak labeling, and dendrite mistargeting, with small meandering projections to incorrect glomeruli. **(D) ***brm*^*-/- *^ventral neuroblast clone PNs exhibit perturbed cell morphology, weak labeling, and a lack of innervation throughout the antennal lobe. **(E) **Dendrites of *Snr1*^*-/- *^DL1 PNs mistarget to non-stereotyped areas of the antennal lobe. **(F, G) ***Snr1*^*-/- *^anterodorsal (F) and lateral (G) neuroblast clone PNs exhibit perturbed cell morphology, weak labeling, and dendrite mistargeting, with small meandering projections to incorrect glomeruli. **(H) ***Snr1*^*-/- *^ventral neuroblast clone PNs exhibit perturbed cell morphology, weak labeling, and a lack of innervation throughout the antennal lobe. Green marks mCD8-GFP-labeled PNs generated by MARCM and labeled using GH146-GAL4. All panels show full confocal stacks; magenta is the presynaptic marker nc82; symbols are as in Figure 2. Scale bar: 20 μm in (A) (for A-H).

In PNs, *brm *mutants displayed anterodorsal single cell clone mistargeting to non-stereotyped glomeruli throughout the antennal lobe, with each clone differing from the next (Figure 5A; n = 16). This is in contrast to the highly stereotyped DA4l mistargeting of *Bap55 *mutants. *brm*^-/- ^neuroblast clones also displayed phenotypes where dendrites make small, meandering projections throughout the antennal lobe, which does not resemble the *Bap55*^*-/- *^phenotype (Figures 5B-D). They additionally exhibit a perturbed cell morphology phenotype, which is markedly more severe than the *Bap55 *mutant phenotype.

We next tested Snr1, a highly conserved subunit of the BRM complex (Figure 1E). It is required to restrict BRM complex activity during the development of wing vein and intervein cells [29] and functions as a growth regulator [30,31]. Its human homolog, SNF5, is strongly correlated with many cancers [32], yet little is known about its specific role in neurons.

In PNs, *Snr1 *mutants displayed similar phenotypes to *brm *mutants. The single cell clones displayed mistargeting to non-stereotyped glomeruli throughout the antennal lobe, with each clone demonstrating a unique phenotype (Figure 5E; n = 31). The neuroblast clones exhibited small meandering dendrites throughout the antennal lobe (Figures 5F-H), which also showed extremely perturbed cell morphology. These results, especially the non-sterotyped single cell clone phenotypes, indicate that the BRM complex functions differently from Bap55 in controlling PN dendrite targeting.

We further analyzed *brm *and *Snr1 *mutants with Mz19-GAL4 to determine whether their phenotypes resembled the *Bap55 *mutant phenotype of derepression. We were unable to generate any labeled clones, indicating one of three possibilities: increased cell death, severe cell proliferation defects, or repression of the Mz19-GAL4 labeling pattern. In any of the three cases, the phenotype does not resemble the *Bap55*^*-/- *^mutant phenotype of abnormal activation of Mz19-GAL4 in single cell or neuroblast clones, indicating that the BRM complex functions differently from Bap55 in PNs.

### *dom *mutant PNs exhibit phenotypes similar to *Bap55 *mutants

**Figure 6:**
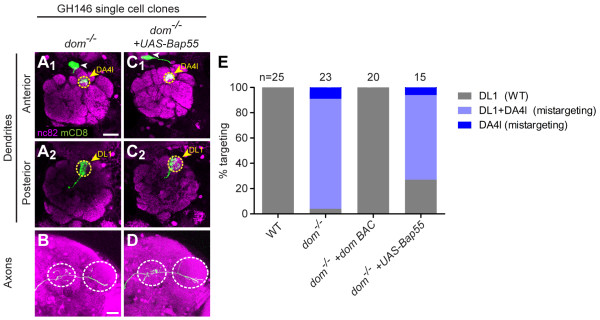
***dom *mutants yield similar phenotypes to *Bap55 *mutants**. **(A, B) ***dom*^*-/- *^DL1 PNs mistarget their dendrites to the anterior glomerulus DA4l (A_1_). *dom*^*-/- *^DL1 PNs retain part of their dendritic mass in the posterior glomerulus DL1 (A_2_). They maintain their L-shaped axon pattern in the lateral horn and branches in the mushroom body calyx (B). **(C, D) **Expression of *UAS-Bap55 *in a *dom*^*-/- *^DL1 PN cannot suppress the dendrite mistargeting phenotype. Dendrites maintain mass in both DA4l (C_1_) and DL1 (C_2_). The axon also maintains its L-shaped pattern (D). **(E) **Quantification of mistargeting phenotypes for (A, C). Green marks mCD8-GFP-labeled PNs generated by MARCM and labeled using GH146-GAL4. (A, C) show partial confocal stacks; (B, D) show full confocal stacks; magenta is the presynaptic marker nc82; symbols are as in Figure 2. Scale bars: 20 μm in (A) (for A, C) and (B) (for B, D).

In the same screen in which we identified the *Bap55 *mutation, we also independently identified LL05537, a mutation in *dom *that resulted in a qualitatively similar phenotype to *Bap55 *mutants. *dom*^-/- ^DL1 PNs split their dendrites between the posterior glomerulus DL1 and the anterior glomerulus DA4l (Figure 6A). Their axons exhibit a WT L-shaped pattern in the lateral horn (Figure 6B).

The LL05537 allele contains a piggyBac insertion in an intron just upstream of the translation start of *dom *(Figure 1F). Because the piggyBac insertion contains splice acceptor sites and stop codons in all three coding frames [22], this allele likely disrupts all isoforms of *dom*. Similarly to *Bap55*, we identified the piggyBac insertion site using inverse PCR [22] and Splinkerette PCR [24]. Precise excision of the piggyBac insertion reverted the dendrite targeting phenotype, confirming that disruption of the *dom *gene causes the dendrite mistargeting (Table 1). In addition, a BAC transgene that contains the entire *dom *transcriptional unit rescued the *dom*^*-/- *^mutant phenotypes (Figure 6E).

Dom is the catalytic DNA-dependent ATPase of the TIP60 complex and has been shown to contribute to a repressive chromatin structure and silencing of homeotic genes. Dom is a member of the SWI/SNF family and its ATPase domain is highly similar to the *Drosophila *Brahma and human BRG1 ATPase domains [20]. The human homolog of Dom is p400, which is important for regulating nucleosome stability during repair of double-stranded DNA breaks [33] and an important regulator of embryonic stem cell identity [34].

To determine whether Bap55 and Dom genetically interact, we expressed *UAS-Bap55 *in a *dom*^-/- ^PN. This manipulation did not significantly alter the dendrite phenotype (Figures 6C, D, quantified in Figure 6E and Table 1; *P *> 0.05 using two-way ANOVA with a Bonferroni posttest comparison across all columns). The axon branching pattern also was not altered.

### *E(Pc) *mutant PNs also exhibit phenotypes similar to *Bap55 *mutants

We also examined another component of the TIP60 complex, E(Pc) (Figure 1G). In *Drosophila*, E(Pc) is a suppressor of position-effect variegation [21,35] and heterozygous mutations in *E(Pc) *result in an increase in homologous recombination over nonhomologous end joining at double-stranded DNA breaks [36]. Following ionizing radiation, heterozygous animals also exhibit higher genome stability and lower incidence of apoptosis [36]. Yet little is known about its role in neurons.

**Figure 7:**
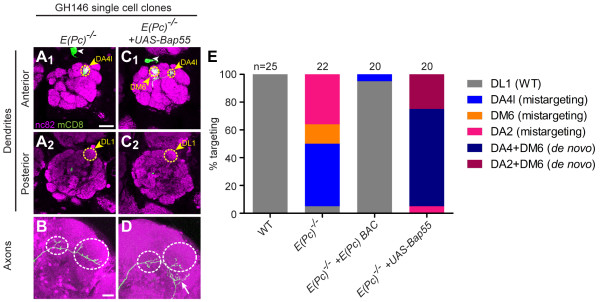
***E(Pc) *mutants yield similar phenotypes to *Bap55 *mutants**. **(A, B) ***E(Pc)*^*-/- *^DL1 PNs mistarget their dendrites to the anterior glomerulus DA4l (A_1_), avoiding the posterior glomerulus DL1 (A_2_), and maintaining the stereotypical L-shaped axon targeting the lateral horn with branches in the mushroom body calyx (B). **(C, D) **Postmitotic expression of *UAS-Bap55 *causes a *de novo *phenotype in *E(Pc)*^*-/- *^DL1 PNs. The dendrites split between DA4l and the anterior medial glomerulus DM6 (C_1_), and the axon mistargets ventrally to the lateral horn (white arrow in (D)). **(E) **Quantification of mistargeting phenotypes for (A, C). Green marks mCD8-GFP-labeled PNs generated by MARCM and labeled using GH146-GAL4. (A, C) show partial confocal stacks; (B, D) show full confocal stacks; magenta is the presynaptic marker nc82; symbols are as in Figure 2. Scale bars: 20 μm in (A) (for A, C) and (B) (for B, D).

In our study, we find that *E(Pc)*^*-/- *^DL1 PN dendrites also mistarget to the anterior glomerulus DA4l (Figure 7A) and exhibit the stereotyped L-shaped axon pattern in the lateral horn (Figure 7B). A BAC transgene that contains the entire *E(Pc) *transcription unit rescued the *E(Pc) *mutant phenotypes (Figure 7E). To determine whether Bap55 and E(Pc) genetically interact, we expressed *UAS-Bap55 *in an *E(Pc)*^-/- ^DL1 PN. This manipulation caused the dendrites to split between the DA4l and DM6 glomeruli (Figure 7C), and resulted in axons targeting ventrally to the lateral horn (Figure 7D, E, Table 1).

### *dom *and *E(Pc) *mutants derepress the expression of a PN-GAL4

**Figure 8:**
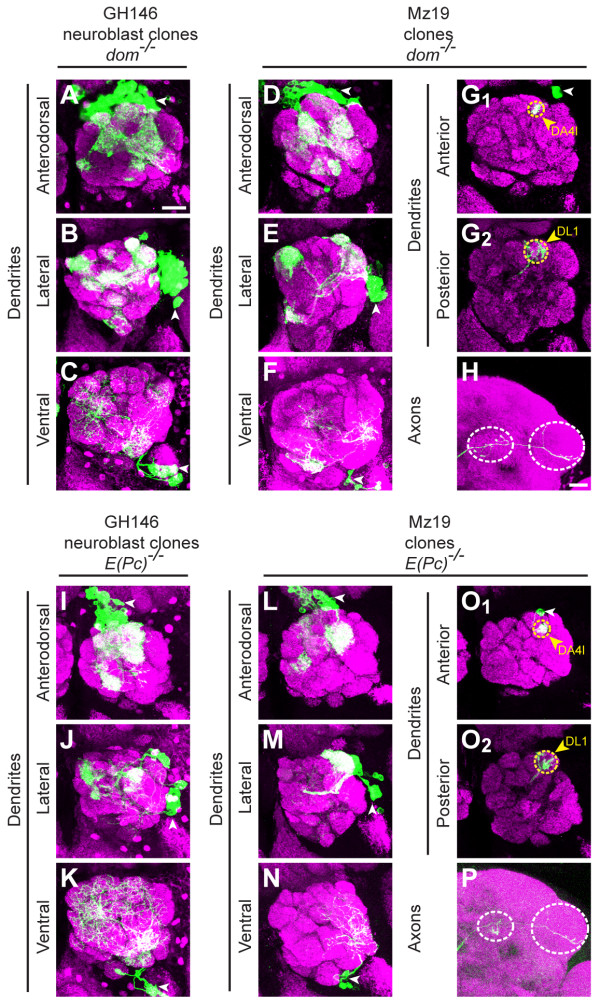
***dom *and *E(Pc) *mutants cause derepression of a PN-GAL4**. **(A, B) ***dom*^*-/- *^GH146-labeled anterodorsal (A) and lateral (B) neuroblast clones show a mild reduction in cell number and disorganization of dendrite targeting. **(C) ***dom*^*-/- *^GH146-labeled ventral neuroblast clones show a mild reduction in dendrite elaboration. **(D, E) ***dom*^*-/- *^Mz19-labeled anterodorsal (D) and lateral (E) neuroblast clones ectopically mark a large number of PNs targeting to many glomeruli. **(F) ***dom*^*-/- *^Mz19-labeled neuroblast clones ectopically mark ventral cells targeting to a number of glomeruli. **(G, H) ***dom*^*-/- *^Mz19 ectopically labels single cell clones, which target dendrites to both DA4l (G_1_) and DL1 (G_2_), with an L-shaped axon pattern in the lateral horn (H). **(I-P) **Equivalent experiments to (A-H) using *E(Pc) *mutants. *E(Pc)*^*-/- *^GH146 and MZ19 clones show similar phenotypes to *dom*^*-/- *^clones. Green marks mCD8-GFP-labeled PNs generated by MARCM and labeled using GH146-GAL4 or Mz19-GAL4. (G, O) show partial confocal stacks; (A-F, H-N, P) show full confocal stacks; magenta is the presynaptic marker nc82; symbols are as in Figure 2. Scale bars: 20 μm in (A) (for A-G, I-O) and (H) (for H, P).

Neuroblast clones mutant for *dom *also exhibit dendrite mistargeting phenotypes to inappropriate glomeruli throughout the antennal lobe. Anterodorsal and lateral neuroblast clones show a very mild reduction in cell number and their dendrites do not target to the full set of proper glomeruli (Figure 8A, B). Ventral neuroblast clones, when compared to WT, exhibit incomplete targeting throughout the antennal lobe (Figure 8C).

Further analysis of *dom *mutants by labeling with Mz19-GAL4 revealed the same derepression as in *Bap55 *mutants (Figure 3). *dom *mutant Mz19-GAL4 PN clones also label anterodorsal, lateral, and ventral neuroblast clones (Figures 8D-F) with phenotypes similar to GH146-GAL4 labeled neuroblast clones (Figures 8A-C). In anterodorsal and lateral neuroblast clones, Mz19-GAL4 labels a large number of PNs that target to many glomeruli throughout the antennal lobe, although the cell number is smaller than GH146-GAL4 labeling (Figures 8D, E). Ventral neuroblast clones are never labeled in WT Mz19-GAL4 (Figure 3C), yet are labeled in *dom *mutants (Figure 8F). Mz19-GAL4 also labels single cell clones that split their dendrites between the DA4l and DL1 glomeruli (Figure 8G) and form the stereotypical L-shaped axon pattern in the lateral horn (Figure 8H). As in *Bap55 *mutants, this compound phenotype likely results from ectopic labeling of a DL1 PN, which further mistargets to DA4l.

The *E(Pc) *phenotypes in GH146 and Mz19-GAL4 labeled neuroblast clones (Figure 8I-N), as well as Mz19-GAL4 labeled single cell clones (Figure 8O, P) displayed similar phenotypes to *dom *as described above (Figure 8A-H). The phenotypic similarities in single cell clone dendrite mistargeting and derepression of a PN-GAL4 in mutations that disrupt *Bap55*, *dom *and *E(Pc) *strongly suggest that these three proteins act together in the TIP60 complex to regulate PN development.

## Discussion

In this study, we demonstrate a similar role for three members of the TIP60 complex in olfactory PN wiring. We find that the TIP60 complex plays a very specific role in controlling dendrite wiring specificity, with a precise mistargeting of the dendrite mass in *Bap55*, *dom*, and *E(Pc) *mutants (Table 1). This specific DL1 to DA4l mistargeting phenotype has only been seen in these three mutants, out of approximately 4,000 other insertional and EMS mutants screened in our laboratory (unpublished data), supporting the conclusion that the TIP60 complex has a specific function in controlling PN dendrite targeting. We find that TIP60 complex mutants show discrete glomerular mistargeting, rather than randomly distributed dendrite spillover to different glomeruli. In contrast, perturbation of individual cell surface receptors often leads to variable mistargeted dendrites that do not necessarily obey glomerular borders [37,38], possibly reflecting the combinatorial use of many cell surface effector molecules. Even transcription factor mutants yield variable phenotypes [5,8]. Interestingly, BRM complex mutants yield non-stereotyped phenotypes in PNs. We do not see a stereotyped glomerular targeting for *brm *or *Snr1 *mutant dendrites; each PN spreads its dendrites across different glomeruli. Our data suggest that different chromatin remodeling complexes play distinct roles in regulating neuronal differentiation. The uni- or bi-glomerular targeting to specific glomeruli implies that the TIP60 complex sits at the top of a regulatory hierarchy to orchestrate an entire transcriptional program of regulation.

Our study suggests a function for Bap55 in *Drosophila *olfactory PN development as a part of the TIP60 complex rather than the BRM complex. Another possibility could be that Bap55 also serves as the interface between the BRM and TIP60 complexes. While loss of core BRM complex components results in a more general defect, loss of Bap55 could specifically disrupt interactions with the TIP60 complex but maintain other BRM complex functions, causing a more specific targeting phenotype mimicking loss of TIP60 complex components.

Interestingly, both human BAF53a and b can significantly rescue the *Bap55*^*-/- *^phenotype. Though in mammals BAF53a is expressed in neural progenitors and BAF53b is expressed in postmitotic neurons [17], they can perform the same postmitotic function in *Drosophila *PNs. Further, both can function with the TIP60 complex in PNs to regulate wiring specificity. These data suggest that the functions for BAF53a and b (in neural precursors and postmitotic neurons, respectively) diverge after the evolutionary split between vertebrates and insects.

The discrete glomerular states of the mistargeting phenotypes may suggest a role for the TIP60 complex upstream of a regulatory hierarchy determining PN targeting decisions. It is possible that disrupting various components changes the composition of the complex. Additionally, overexpression of Bap55 in various mutant backgrounds might alter the sensitive stoichiometry of the TIP60 complex, resulting in targeting to different but still distinct glomeruli.

Studies in our laboratory have identified several mutants that cause DL1 PNs to mistarget to areas near the DM6 glomerulus [8] (Table 1 and unpublished results). Interestingly, WT DM6 PNs have the most similar lateral horn axon arborization pattern to DL1 PNs [2]. We hypothesize that the transcriptional code for DM6 is similar to that of DL1, which is at least partially regulated by the TIP60 complex. The genes described in this manuscript are the only mutants that have yielded specific DA4l mistargeting to date. It is possible that the targeting 'code' for DA4l, DL1, and DM6 may be most similar, such that perturbation of the TIP60 complex might result in reprogramming of dendrite targeting. PNs have previously been shown to be pre-specified by birth order [1]. Yet DA4l is born in early embryogenesis, DL1 is born in early larva, and DM6 is born in late larva [39]. This implies that the TIP60 transcriptional code does not correlate with PN birth order. The mechanisms by which the TIP60 complex specifies PN dendrite targeting remain to be determined.

## Conclusions

In this study, we characterize PN phenotypes of mutants in the BRM and TIP60 complexes, with a focus on Bap55, which is shared by the two complexes. We find that the TIP60 complex plays a very specific role in regulating PN dendrite targeting; mutants mistarget from the DL1 to the DA4l glomerulus. This specific mistargeting phenotype suggests that TIP60 controls a transcriptional program important for making dendrite targeting decisions.

## Materials and methods

### Fly stocks

A MARCM-based mosaic screen was performed using piggyBac insertional mutants previously described and currently available at the Kyoto *Drosophila *Genetic Resource Center [22]. The insertion sites of piggyBac mutants in *Bap55 *(LL05955) and *domino *(LL05537) were confirmed by inverse PCR [22], precise excision, and Splinkerette PCR [24].

The *brm*^*2*^, *Snr1*^*01319*^, and *E(Pc)*^*1 *^alleles were obtained from the Bloomington *Drosophila *Stock Center and *Snr1*^*6C *^was kindly provided by JA Simon [40]. We recombined the alleles onto FRT-containing chromosomes using standard techniques.

The *GH146-GAL4 *transgene has been previously described [41]. An insertion on the fourth chromosome was used in all MARCM experiments on 2R (Figures 2, 4, 6, 7, 8A-C, I-K).

### Immunostaining

MARCM was performed as described and flies were raised at 25°C [42]. Fly brains of both genders were dissected, fixed, and stained as described [43]. Antibody conditions: rat anti-mCD8α 1:100 (Invitrogen Caltag #RM2200 or #MCD0800, Carlsbad, CA, USA), mouse anti-nc82 1:40 (Developmental Studies Hybridoma Bank #nc82, contributed by E Buchner, Iowa City, IA, USA).

### Construction of *UAS-Bap55*

To generate *UAS-Bap55*, a full length cDNA (LD29458, Berkeley *Drosophila *Genome Project Gold cDNA, *Drosophila *Genomics Resource Center, Bloomington, IN, USA) was amplified using the following primers (5'-3'): CACCCAAAATGAGTGGCGGCACCATGCTATATG and TTACGGACACTTCCGCTCGACTTGG. The first primer amplifies from the 5' end and adds a CACC overhang for the TOPO reaction and a Kozak sequence. The second primer amplifies to the stop codon. The PCR product was subcloned into *pENTR-D/TOPO *(Invitrogen) and recombined into the destination vector *pUAST-Gateway-attB *[8] using LR Clonase II (Invitrogen).

The final construct was integrated into the 86Fb landing site [44] on the third chromosome.

### Construction of *UAS-BAF53a *and *UAS-BAF53b*

To generate *UAS-BAF53a *and *UAS-BAF53b*, full length cDNAs (kind gifts of GR Crabtree [17]) were amplified using the following primers (5'-3'): for *UAS-BAF53a*, CACCCAAAATGAGCGGCGGCGTGTACGGCG and TCAAGGGCACTTTCTTTCTACACACTG; for *UAS-BAF53b*, CACCCAAAATGAGCGGGGGCGTCTACGGCG and TCAGGGGCACTTCCGCTCCACGCACTG. The first primer amplifies from the 5' end and adds a CACC overhang for the TOPO reaction and a Kozak sequence. The second primer amplifies to the stop codon. The PCR product was subcloned into *pENTR-D/TOPO *(Invitrogen) and recombined into the destination vector *pUAST-Gateway-attB *[8] using LR Clonase II (Invitrogen).

The final construct was integrated into the 86Fb landing site [44] on the third chromosome.

### Construction of BAC transgenic flies containing *dom *or *E(Pc)*

To generate BAC transgenic flies containing *dom*, BAC #CH321-01P07 in the attB-P[acman]-CmR vector from the BACPAC Resources Center (Oakland, CA, USA) was verified and sent to BestGene, Inc. (Chino Hills, CA, USA) for integration into the 86Fb landing site [44] on the third chromosome.

BAC transgenic flies containing *E(Pc) *were generated in the same manner, using BAC #CH322-140G22.

## Abbreviations

BAC: bacterial artificial chromosome; BAF: Brg1 associated factors; Bap55: Brahma associated protein 55kD; BRM: Brahma; Dom: Domino; E(Pc): Enhancer of Polycomb; GFP: green fluorescent protein; PN: projection neuron; WT: wild type.

## Competing interests

The authors declare that they have no competing interests.

## Authors' contributions

JST carried out all the experiments. JST and LL designed the study and wrote the manuscript.
